# Supplementary material for: Public participation in crisis policymaking. How 30,000 Dutch citizens advised their government on relaxing COVID-19 lockdown measures
Source: PLoS One. 2021 May 6;16(5):e0250614. doi: 10.1371/journal.pone.0250614 (PMC8101923; doi:10.1371/journal.pone.0250614)
Supplement: S1 Appendix — (DOCX) [file pone.0250614.s001.docx]

**S1 Appendix: Experimental design process.**

The first part of the experimental design consisted of defining the possible impact levels and pressure on the healthcare system of each relaxation option, based on the feedback and information that was obtained in the PVE design process. S1 Table 1 summarizes each possible impact including the increase in the pressure on the health care system caused by the relaxation option.

S1 Table 1: Possible impact levels and pressure on the healthcare system for each relaxation option.

| **Relaxation option** | **Pressure on the healthcare system** | **Additional deaths of people of +70 years** | **Additional deaths of people of less than 70 years** | **Additional people with permanent physical injury** | **Minus people with permanent mental injury** | **Minus households that have lost 15% of income** |
| --- | --- | --- | --- | --- | --- | --- |
| 1: Nursing and care homes allow visitors | 10%  15%  20%  25% | 1500  2000  3000 | 30  50  100  150  300 | 100  500  1000 | 30000  60000 | 50  200 |
| 2: Re-open businesses (other than contact professions and hospitality industry) | 6%  8%  10%  15% | 200  400  600  1000 | 150  300  500  750 | 1000  2000  3000  5000  7500 | 1000  2000  5000  7500 | 10000  20000  50000  75000 |
| 3: Re-open contact professions | 8%  10%  15% | 200  400  600  1000 | 150  300  500  750,  1000 | 1000  2000  3000  5000  7500  10000 | 5000  7500  10000  15000 | 20000  50000  75000 |
| 4: Young people may come together in small groups | 4%  6%  8% | 50  200  400 | 50  100  150  300 | 500  1000  2000  3000  5000 | 2000  5000  7500  10000  15000 | 50  200  5000 |
| 5: All restrictions lifted for people with immunity | 10%  15%  20% | 400  600  1000  1500 | 300  500  750 | 2000  3000  5000 | 1000  2000  5000  7500 | 5000  10000  20000 |
| 6: All restrictions lifted in Northern provinces | 15%  20%  25%  30% | 600  1000  1500  2000 | 300  500  750  1000 | 5000  7500  10000 | 10000  15000  30000 | 20000  50000  75000 |
| 7: Direct family members from other households can have social contact | 6%  8%  10%  15% | 600  1000  1500  2000 | 300  500  750  1000 | 2000  3000  5000  7500  10000 | 30000  60000 | 50 |
| 8: Re-open hospitality and entertainment industry | 15%  20%  25% | 200  400  600  1000 | 300  500  750  1000 | 1000  2000  3000  5000  7500  10000 | 15000  30000  60000 | 50000  75000  100000 |

Ideally, an experimental design should consider all possible combinations of impact and pressure levels, in order to capture information from all the possible profiles of relaxation strategies from participants choices in the PVE. This is called in literature as a “full-factorial design”. However, collecting data for such design is often non-tractable, because the number of combinations explodes even for small numbers of relaxation strategies, impacts, and impact/pressure levels. For this PVE, a full factorial design is composed of more than $1.59*{10}^{26}$ combinations.

There are several solutions to reduce the number of combinations of the experimental design. The first and most intuitive one is to take a random number of profiles (defined by the researcher) of the full factorial design. This is called in literature as a “fractional factorial design”. A problem of this approach is that it artificially increases the correlation level between impact/pressure levels of the experimental design. In turn, this increased correlation has an impact on the possibility of extracting information related to preferences for impacts (i.e. taste parameters) using econometric models. In light of this, the construction of a reduced experimental design should ensure that the correlation between attributes is minimal.

The experimental design of this PVE aims to obtain a tractable number of profiles, at the same time that the correlation between impact/pressure levels is minimized. In particular, we aim to minimize the maximum value of the correlation matrix between impact/pressure levels of the design:

| $D^{*}=\arg\min\left[ \rho_{MAX} \right],$ | $\rho_{MAX}=max \left( Corr\left( \boldsymbol{X},\boldsymbol{C} \right) \right)$ |
| --- | --- |

Where $D^{*}$ is the optimal design matrix, $\boldsymbol{X}$ is a $N\times J\times K$ matrix of impact levels for $N$ profiles, $J$ relaxation options, and $K$ impacts; $\boldsymbol{C}$ is a $N\times J$ matrix of pressure levels for each profile and relaxation option. We call this type of designs as “min-max correlation” designs.

We developed an algorithm that creates min-max correlation designs by iteratively selecting impact/pressure levels, evaluating on each step whether the max-correlation is reduced. This algorithm is described as follows:

- Step 0 (Definition of inputs): Define the set of possible impact and pressure levels for each relaxation option. Define $N$ as the number of required profiles.
- Step 1 (Definition of initial candidate design): Construct 10 designs with N profiles each one, by taking random levels of impact and pressure levels from the set defined on Step 0. Keep the design with the smallest max-correlation value and call it as “(initial) candidate design”.
- Step 2 (Replacement): Set a random impact/pressure from the candidate design, and replace its value with a random impact/pressure value from the set defined in Step 0, and call it as the new “candidate design”.
- Step 3 (Evaluation): Compute the max-correlation value of the candidate design.
- Step 4 (Decision): If the max-correlation value of the new candidate design is reduced, the change is kept and go back to Step 2. Otherwise, the change is reverted and go back to Step 2.

This algorithm is conducted until a certain number of iterations without improvement is reached, or until a certain amount of time. Then, the last stored design is called as the optimal design. For this PVE, we ran the algorithm for 10 minutes, although we observed no further improvement after 3 minutes approximately. Finally, we introduced additional constraints to the replacement step in the algorithm in order to avoid that a possible impact/pressure level does not appear in the optimal design.
