# Supplementary material for: Public participation in crisis policymaking. How 30,000 Dutch citizens advised their government on relaxing COVID-19 lockdown measures
Source: PLoS One. 2021 May 6;16(5):e0250614. doi: 10.1371/journal.pone.0250614 (PMC8101923; doi:10.1371/journal.pone.0250614)
Supplement: S2 Appendix — (DOCX) [file pone.0250614.s002.docx]

**S2 Appendix: Descriptive information for sociodemographic variables used in Fig 2 and Table 4**

S2 Table 1: Frequency of sociodemographic variables used in Fig 2 and Table 4

| **Gender** | **Open sample** | **Representative sample** |
| --- | --- | --- |
| Men | 10425 (49.21%) | 1447 (51.35%) |
| Women | 10705 (50.53%) | 1365 (48.44%) |
| Other | 56 (0.26%) | 6 (0.21%) |
| No answer | 5107 | 540 |
| **Age group** |  |  |
| 18-25 yr. | 1894 (8.94%) | 483 (17.14%) |
| 26-35 yr. | 3915 (18.48%) | 430 (15.26%) |
| 36-45 yr. | 3617 (17.07%) | 527 (18.7%) |
| 46-55 yr. | 4749 (22.42%) | 416 (14.76%) |
| 56-65 yr. | 4266 (20.14%) | 360 (12.78%) |
| 66-74 yr. | 2312 (10.91%) | 455 (16.15%) |
| 75+ yr. | 433 (2.04%) | 147 (5.22%) |
| No answer | 5107 | 540 |
| **Maximum education level** |  |  |
| No education | 25 (0.12%) | 20 (0.71%) |
| Primary school | 42 (0.2%) | 269 (9.55%) |
| Primary vocational school | 183 (0.86%) | 666 (23.63%) |
| Secondary vocational school | 663 (3.13%) | 410 (14.55%) |
| High school | 1171 (5.53%) | 33 (1.17%) |
| Junior college | 2221 (10.48%) | 174 (6.17%) |
| University of applied sciences | 7925 (37.41%) | 321 (11.39%) |
| University | 8956 (42.27%) | 925 (32.82%) |
| No answer | 5107 | 540 |
| **Province** |  |  |
| Groningen | 564 (2.66%) | 102 (3.62%) |
| Friesland | 402 (1.9%) | 157 (5.57%) |
| Drenthe | 358 (1.69%) | 339 (12.03%) |
| Overijssel | 2307 (10.89%) | 431 (15.29%) |
| Flevoland | 731 (3.45%) | 65 (2.31%) |
| Gelderland | 1908 (9.01%) | 218 (7.74%) |
| Utrecht | 2550 (12.04%) | 143 (5.07%) |
| Noord-Holland | 3397 (16.03%) | 76 (2.7%) |
| Zuid-Holland | 6019 (28.41%) | 83 (2.95%) |
| Zeeland | 228 (1.08%) | 225 (7.98%) |
| Noord-Brabant | 1991 (9.4%) | 567 (20.12%) |
| Limburg | 731 (3.45%) | 412 (14.62%) |
| No answer | 5107 | 540 |
| **Perceived risk of becoming very ill** |  |  |
| No risk | 531 (2.46%) | 950 (33.12%) |
| Low risk | 8354 (38.74%) | 594 (20.71%) |
| Moderate risk | 8606 (39.91%) | 92 (3.21%) |
| High risk | 3347 (15.52%) | 1017 (35.46%) |
| Extreme risk | 725 (3.36%) | 215 (7.5%) |
| No answer | 4730 | 490 |
| **Total sample** | **26293** | **3358** |
