# Supplementary material for: Public participation in crisis policymaking. How 30,000 Dutch citizens advised their government on relaxing COVID-19 lockdown measures
Source: PLoS One. 2021 May 6;16(5):e0250614. doi: 10.1371/journal.pone.0250614 (PMC8101923; doi:10.1371/journal.pone.0250614)
Supplement: S3 Appendix — (DOCX) [file pone.0250614.s003.docx]

**S3 Appendix: MDCEV estimates and optimal portfolio for separate samples**

S3 Table 1: MDCEV model estimates. Separate samples

|  | **Open sample** | **Representative sample** |
| --- | --- | --- |
| **Policy-specific constants:** |  |  |
| 1: Nursing and care homes allow visitors | 2.6865^***^ | 2.7219^***^ |
|  | (0.0297) | (0.0764) |
| 2: Re-open businesses (other than contact professions and hospitality industry) | 2.6451^***^ | 2.4132^***^ |
|  | (0.0233) | (0.0556) |
| 3: Re-open contact professions | 3.2382^***^ | 2.8500^***^ |
|  | (0.0276) | (0.0631) |
| 4: Young people may come together in small groups | 1.8825^***^ | 1.6317^***^ |
|  | (0.0142) | (0.0357) |
| 5: All restrictions lifted for people with immunity | 1.5608^***^ | 1.9986^***^ |
|  | (0.0211) | (0.0533) |
| 6: All restrictions lifted in Northern provinces | 1.5954^***^ | 2.0641^***^ |
|  | (0.0342) | (0.0809) |
| 7: Direct family members from other households can have social contact | 2.4893^***^ | 2.6784^***^ |
|  | (0.0294) | (0.0748) |
| 8: Re-open hospitality and entertainment industry | 2.7346^***^ | 2.4078^***^ |
|  | (0.0376) | (0.0857) |
| **Taste parameters:** |  |  |
| Additional 10.000 deaths of people of +70 years | -0.4123^***^ | -1.1009^***^ |
|  | (0.0945) | (0.2308) |
| Additional 10.000 deaths of people of less than 70 years | -0.9295^***^ | -0.4503 |
|  | (0.1933) | (0.4380) |
| Additional 10.000 people with permanent physical injury | -0.1033^***^ | -0.1481^***^ |
|  | (0.0174) | (0.0434) |
| Minus 10.000 people with permanent mental injury | 0.0023 | -0.0121 |
|  | (0.0037) | (0.0094) |
| Minus 10.000 households that have lost 15% of income | 0.0094^***^ | -0.0042 |
|  | (0.0026) | (0.0060) |
| Observations | 26293 | 3358 |
| Log-likelihood | -127928.8123 | -16499.3413 |
| AIC | 255831.6246 | 32972.6826 |
| BIC | 255725.3229 | 32893.1343 |
| **Note:** Standard errors in parenthesis. **Statistical significance:** ^***^p < 0.001, ^**^p < 0.01, ^*^p < 0.05 | | |

S3 Table 1: Optimal portfolios of relaxation options. Separate samples

|  | **Open sample** | | | **Representative sample** | | |
| --- | --- | --- | --- | --- | --- | --- |
|  | Avg. | Pessim. | Optim | Avg. | Pessim. | Optim |
| 1: Nursing and care homes allow visitors |  |  | X |  |  | X |
| 2: Re-open businesses (other than contact professions and hospitality industry) | X |  | X |  |  | X |
| 3: Re-open contact professions | X | X | X | X |  | X |
| 4: Young people may come together in small groups |  |  | X |  |  | X |
| 5: All restrictions lifted for people with immunity |  |  |  |  |  |  |
| 6: All restrictions lifted in Northern provinces |  |  |  |  |  |  |
| 7: Direct family members from other households can have social contact | X |  | X | X |  | X |
| 8: Re-open hospitality and entertainment industry |  |  | X |  |  |  |
| **Pressure to the healthcare system** | **31.6%** | **15%** | **49%** | **21.8%** | **0%** | **34%** |
