# Supplementary material for: Public participation in crisis policymaking. How 30,000 Dutch citizens advised their government on relaxing COVID-19 lockdown measures
Source: PLoS One. 2021 May 6;16(5):e0250614. doi: 10.1371/journal.pone.0250614 (PMC8101923; doi:10.1371/journal.pone.0250614)
Supplement: S4 Appendix — Sample of provinces of Friesland, Groningen and Drenthe (the Northern provinces). (DOCX) [file pone.0250614.s004.docx]

**S4 Appendix: Quantitative results and impact/pressure levels used for sensitivity analysis. Sample of provinces of Friesland, Groningen and Drenthe (the Northern provinces)**

S4 Table 1: MDCEV model estimates. Sample for individuals who live in the Northern provinces of Friesland, Groningen and Drenthe

|  | **Estimates** |
| --- | --- |
| **Baseline utility of relaxation strategies:** |  |
| 1: Nursing and care homes allow visitors | 2.7526^***^ |
|  | (0.1085) |
| 2: Re-open businesses (other than contact professions and hospitality industry) | 2.3516^***^ |
|  | (0.0797) |
| 3: Re-open contact professions | 2.9320^***^ |
|  | (0.0923) |
| 4: Young people may come together in small groups | 1.7161^***^ |
|  | (0.0511) |
| 5: All restrictions lifted for people with immunity | 1.4744^***^ |
|  | (0.0803) |
| 6: All restrictions lifted in Northern provinces | 2.2522^***^ |
|  | (0.1139) |
| 7: Direct family members from other households can have social contact | 2.3574^***^ |
|  | (0.1072) |
| 8: Re-open hospitality and entertainment industry | 2.4172^***^ |
|  | (0.1219) |
| **Impact effects:** |  |
| Additional 10.000 deaths of people of +70 years | -0.7926^*^ |
|  | (0.3298) |
| Additional 10.000 deaths of people of less than 70 years | -0.7957 |
|  | (0.6428) |
| Additional 10.000 people with permanent physical injury | -0.0492 |
|  | (0.0616) |
| Minus 10.000 people with permanent mental injury | 0.0042 |
|  | (0.0132) |
| Minus 10.000 households that have lost 15% of income | 0.0007 |
|  | (0.0085) |
| Observations | 1645 |
| Log-likelihood | -8073.5348 |
| AIC | 16121.0695 |
| BIC | 16050.7981 |

S4 Table 2: Optimal portfolios of relaxation options. Sample for individuals who live in the Northern provinces of Friesland, Groningen and Drenthe

|  | **Averages** | **Pessimistic** | **Optimistic** |
| --- | --- | --- | --- |
| 1: Nursing and care homes allow visitors |  |  | X |
| 2: Re-open businesses (other than contact professions and hospitality industry) |  |  | X |
| 3: Re-open contact professions | X | X | X |
| 4: Young people may come together in small groups |  |  | X |
| 5: All restrictions lifted for people with immunity |  |  |  |
| 6: All restrictions lifted in Northern provinces |  |  |  |
| 7: Direct family members from other households can have social contact |  |  | X |
| 8: Re-open hospitality and entertainment industry |  |  |  |
| **Pressure to the healthcare system** | **11.4%** | **15%** | **34%** |

S4 Table 3: Impact levels used for optimal portfolio computation for three scenarios. Sample for individuals who live in the Northern provinces of Friesland, Groningen and Drenthe

| **Impact** | **Relaxation strategy** | **Average** | **Conservative** | **Optimistic** |
| --- | --- | --- | --- | --- |
| Additional deaths of people of +70 years | Nursing and care homes allow visitors | 2185.41 | 3000 | 1500 |
|  | Re-open businesses (other than contact professions and hospitality industry) | 712.58 | 1000 | 200 |
|  | Re-open contact professions | 591.85 | 1000 | 200 |
|  | Young people may come together in small groups | 251.52 | 400 | 50 |
|  | All restrictions lifted for people with immunity | 1060.49 | 1500 | 400 |
|  | All restrictions lifted in Northern provinces | 1218.18 | 2000 | 600 |
|  | Direct family members from other households can have social contact | 1127.54 | 2000 | 600 |
|  | Re-open hospitality and entertainment industry | 567.29 | 1000 | 200 |
| Additional deaths of people of less than 70 years | Nursing and care homes allow visitors | 130.70 | 300 | 30 |
|  | Re-open businesses (other than contact professions and hospitality industry) | 468.33 | 750 | 150 |
|  | Re-open contact professions | 576.66 | 1000 | 150 |
|  | Young people may come together in small groups | 161.25 | 300 | 50 |
|  | All restrictions lifted for people with immunity | 554.89 | 750 | 300 |
|  | All restrictions lifted in Northern provinces | 641.82 | 1000 | 300 |
|  | Direct family members from other households can have social contact | 605.74 | 1000 | 300 |
|  | Re-open hospitality and entertainment industry | 570.67 | 1000 | 300 |
| Additional people with permanent physical injury | Nursing and care homes allow visitors | 619.88 | 1000 | 100 |
|  | Re-open businesses (other than contact professions and hospitality industry) | 4049.24 | 7500 | 1000 |
|  | Re-open contact professions | 4612.16 | 10000 | 1000 |
|  | Young people may come together in small groups | 2636.17 | 5000 | 500 |
|  | All restrictions lifted for people with immunity | 3430.40 | 5000 | 2000 |
|  | All restrictions lifted in Northern provinces | 7500 | 10000 | 5000 |
|  | Direct family members from other households can have social contact | 5422.80 | 10000 | 2000 |
|  | Re-open hospitality and entertainment industry | 4859.27 | 10000 | 1000 |
| Reduction of people with permanent mental injury | Nursing and care homes allow visitors | 41069.91 | 30000 | 60000 |
|  | Re-open businesses (other than contact professions and hospitality industry) | 4031 | 1000 | 7500 |
|  | Re-open contact professions | 9575.99 | 5000 | 15000 |
|  | Young people may come together in small groups | 6765.96 | 2000 | 10000 |
|  | All restrictions lifted for people with immunity | 3676.60 | 1000 | 7500 |
|  | All restrictions lifted in Northern provinces | 17054.71 | 10000 | 30000 |
|  | Direct family members from other households can have social contact | 46778.12 | 30000 | 60000 |
|  | Re-open hospitality and entertainment industry | 42246.20 | 15000 | 60000 |
| Reduction of households that have lost 15% of income | Nursing and care homes allow visitors | 141.19 | 50 | 200 |
|  | Re-open businesses (other than contact professions and hospitality industry) | 38270.52 | 10000 | 75000 |
|  | Re-open contact professions | 50434.65 | 20000 | 75000 |
|  | Young people may come together in small groups | 1371.37 | 50 | 5000 |
|  | All restrictions lifted for people with immunity | 12167.17 | 5000 | 20000 |
|  | All restrictions lifted in Northern provinces | 50206.69 | 20000 | 75000 |
|  | Direct family members from other households can have social contact | 50 | 50 | 50 |
|  | Re-open hospitality and entertainment industry | 75516.72 | 50000 | 100000 |

S4 Table 4: Pressure to the healthcare system used for optimal portfolio computation for three scenarios. Sample for individuals who live in the Northern provinces of Friesland, Groningen and Drenthe

| **Relaxation strategy** | **Average** | **Conservative** | **Optimistic** |
| --- | --- | --- | --- |
| Nursing and care homes allow visitors | 18.02 | 25 | 10 |
| Re-open businesses (other than contact professions and hospitality industry) | 9.87 | 15 | 6 |
| Re-open contact professions | 11.49 | 15 | 8 |
| Young people may come together in small groups | 6.59 | 8 | 4 |
| All restrictions lifted for people with immunity | 15.19 | 20 | 10 |
| All restrictions lifted in Northern provinces | 22.23 | 30 | 15 |
| Direct family members from other households can have social contact | 10.11 | 15 | 6 |
| Re-open hospitality and entertainment industry | 19.09 | 25 | 15 |
