# Supplementary material for: Public participation in crisis policymaking. How 30,000 Dutch citizens advised their government on relaxing COVID-19 lockdown measures
Source: PLoS One. 2021 May 6;16(5):e0250614. doi: 10.1371/journal.pone.0250614 (PMC8101923; doi:10.1371/journal.pone.0250614)
Supplement: S5 Appendix — (DOCX) [file pone.0250614.s005.docx]

**S5 Appendix: Impact/pressure levels used for sensitivity analysis, for each type of sample.**

S5 Table 1: Impact levels used for optimal portfolio computation for three scenarios. Open sample

| **Impact** | **Relaxation strategy** | **Average** | **Conservative** | **Optimistic** |
| --- | --- | --- | --- | --- |
| Additional deaths of people of +70 years | Nursing and care homes allow visitors | 2215.27 | 3000 | 1500 |
|  | Re-open businesses (other than contact professions and hospitality industry) | 714.07 | 1000 | 200 |
|  | Re-open contact professions | 593.74 | 1000 | 200 |
|  | Young people may come together in small groups | 248.61 | 400 | 50 |
|  | All restrictions lifted for people with immunity | 1063.01 | 1500 | 400 |
|  | All restrictions lifted in Northern provinces | 1220.99 | 2000 | 600 |
|  | Direct family members from other households can have social contact | 1120.51 | 2000 | 600 |
|  | Re-open hospitality and entertainment industry | 569.73 | 1000 | 200 |
| Additional deaths of people of less than 70 years | Nursing and care homes allow visitors | 129.62 | 300 | 30 |
|  | Re-open businesses (other than contact professions and hospitality industry) | 471.64 | 750 | 150 |
|  | Re-open contact professions | 573.65 | 1000 | 150 |
|  | Young people may come together in small groups | 161.98 | 300 | 50 |
|  | All restrictions lifted for people with immunity | 548.56 | 750 | 300 |
|  | All restrictions lifted in Northern provinces | 638.18 | 1000 | 300 |
|  | Direct family members from other households can have social contact | 597.84 | 1000 | 300 |
|  | Re-open hospitality and entertainment industry | 569.27 | 1000 | 300 |
| Additional people with permanent physical injury | Nursing and care homes allow visitors | 614.30 | 1000 | 100 |
|  | Re-open businesses (other than contact professions and hospitality industry) | 3961.32 | 7500 | 1000 |
|  | Re-open contact professions | 4611.11 | 10000 | 1000 |
|  | Young people may come together in small groups | 2592.76 | 5000 | 500 |
|  | All restrictions lifted for people with immunity | 3404.78 | 5000 | 2000 |
|  | All restrictions lifted in Northern provinces | 7522.15 | 10000 | 5000 |
|  | Direct family members from other households can have social contact | 5540.20 | 10000 | 2000 |
|  | Re-open hospitality and entertainment industry | 4945.23 | 10000 | 1000 |
| Reduction of people with permanent mental injury | Nursing and care homes allow visitors | 40728.71 | 30000 | 60000 |
|  | Re-open businesses (other than contact professions and hospitality industry) | 4059.84 | 1000 | 7500 |
|  | Re-open contact professions | 9532.19 | 5000 | 15000 |
|  | Young people may come together in small groups | 6756.25 | 2000 | 10000 |
|  | All restrictions lifted for people with immunity | 3721.41 | 1000 | 7500 |
|  | All restrictions lifted in Northern provinces | 17249.46 | 10000 | 30000 |
|  | Direct family members from other households can have social contact | 46466.74 | 30000 | 60000 |
|  | Re-open hospitality and entertainment industry | 42606.21 | 15000 | 60000 |
| Reduction of households that have lost 15% of income | Nursing and care homes allow visitors | 140.75 | 50 | 200 |
|  | Re-open businesses (other than contact professions and hospitality industry) | 39210.82 | 10000 | 75000 |
|  | Re-open contact professions | 49987.83 | 20000 | 75000 |
|  | Young people may come together in small groups | 1408.73 | 50 | 5000 |
|  | All restrictions lifted for people with immunity | 12051.50 | 5000 | 20000 |
|  | All restrictions lifted in Northern provinces | 49546.08 | 20000 | 75000 |
|  | Direct family members from other households can have social contact | 50 | 50 | 50 |
|  | Re-open hospitality and entertainment industry | 75771.12 | 50000 | 100000 |

S5 Table 2: Pressure to the healthcare system used for optimal portfolio computation for three scenarios. Open sample

| **Relaxation strategy** | **Average** | **Conservative** | **Optimistic** |
| --- | --- | --- | --- |
| Nursing and care homes allow visitors | 17.92 | 25 | 10 |
| Re-open businesses (other than contact professions and hospitality industry) | 9.87 | 15 | 6 |
| Re-open contact professions | 11.51 | 15 | 8 |
| Young people may come together in small groups | 6.54 | 8 | 4 |
| All restrictions lifted for people with immunity | 15.15 | 20 | 10 |
| All restrictions lifted in Northern provinces | 22.25 | 30 | 15 |
| Direct family members from other households can have social contact | 10.22 | 15 | 6 |
| Re-open hospitality and entertainment industry | 18.97 | 25 | 15 |

S5 Table 3: Impact levels used for optimal portfolio computation for three scenarios. Representative sample

| **Impact** | **Relaxation strategy** | **Average** | **Conservative** | **Optimistic** |
| --- | --- | --- | --- | --- |
| Additional deaths of people of +70 years | Nursing and care homes allow visitors | 2223.65 | 3000 | 1500 |
|  | Re-open businesses (other than contact professions and hospitality industry) | 706.37 | 1000 | 200 |
|  | Re-open contact professions | 588.92 | 1000 | 200 |
|  | Young people may come together in small groups | 250.07 | 400 | 50 |
|  | All restrictions lifted for people with immunity | 1062.63 | 1500 | 400 |
|  | All restrictions lifted in Northern provinces | 1221.11 | 2000 | 600 |
|  | Direct family members from other households can have social contact | 1110.75 | 2000 | 600 |
|  | Re-open hospitality and entertainment industry | 570.40 | 1000 | 200 |
| Additional deaths of people of less than 70 years | Nursing and care homes allow visitors | 129.01 | 300 | 30 |
|  | Re-open businesses (other than contact professions and hospitality industry) | 471.86 | 750 | 150 |
|  | Re-open contact professions | 569.57 | 1000 | 150 |
|  | Young people may come together in small groups | 161.49 | 300 | 50 |
|  | All restrictions lifted for people with immunity | 549.24 | 750 | 300 |
|  | All restrictions lifted in Northern provinces | 639.90 | 1000 | 300 |
|  | Direct family members from other households can have social contact | 597.29 | 1000 | 300 |
|  | Re-open hospitality and entertainment industry | 569.88 | 1000 | 300 |
| Additional people with permanent physical injury | Nursing and care homes allow visitors | 604.05 | 1000 | 100 |
|  | Re-open businesses (other than contact professions and hospitality industry) | 4010.42 | 7500 | 1000 |
|  | Re-open contact professions | 4625.67 | 10000 | 1000 |
|  | Young people may come together in small groups | 2580.85 | 5000 | 500 |
|  | All restrictions lifted for people with immunity | 3410.66 | 5000 | 2000 |
|  | All restrictions lifted in Northern provinces | 7510.42 | 10000 | 5000 |
|  | Direct family members from other households can have social contact | 5498.66 | 10000 | 2000 |
|  | Re-open hospitality and entertainment industry | 4864.35 | 10000 | 1000 |
| Reduction of people with permanent mental injury | Nursing and care homes allow visitors | 40640.26 | 30000 | 60000 |
|  | Re-open businesses (other than contact professions and hospitality industry) | 4032.61 | 1000 | 7500 |
|  | Re-open contact professions | 9586.81 | 5000 | 15000 |
|  | Young people may come together in small groups | 6745.09 | 2000 | 10000 |
|  | All restrictions lifted for people with immunity | 3725.58 | 1000 | 7500 |
|  | All restrictions lifted in Northern provinces | 17374.93 | 10000 | 30000 |
|  | Direct family members from other households can have social contact | 46340.08 | 30000 | 60000 |
|  | Re-open hospitality and entertainment industry | 42480.64 | 15000 | 60000 |
| Reduction of households that have lost 15% of income | Nursing and care homes allow visitors | 140.54 | 50 | 200 |
|  | Re-open businesses (other than contact professions and hospitality industry) | 38941.33 | 10000 | 75000 |
|  | Re-open contact professions | 50064.03 | 20000 | 75000 |
|  | Young people may come together in small groups | 1466.24 | 50 | 5000 |
|  | All restrictions lifted for people with immunity | 12182.85 | 5000 | 20000 |
|  | All restrictions lifted in Northern provinces | 50023.82 | 20000 | 75000 |
|  | Direct family members from other households can have social contact | 50 | 50 | 50 |
|  | Re-open hospitality and entertainment industry | 75707.27 | 50000 | 100000 |

S5 Table 4: Pressure to the healthcare system used for optimal portfolio computation for three scenarios. Representative sample

| **Relaxation strategy** | **Average** | **Conservative** | **Optimistic** |
| --- | --- | --- | --- |
| Nursing and care homes allow visitors | 17.99 | 25 | 10 |
| Re-open businesses (other than contact professions and hospitality industry) | 9.91 | 15 | 6 |
| Re-open contact professions | 11.49 | 15 | 8 |
| Young people may come together in small groups | 6.52 | 8 | 4 |
| All restrictions lifted for people with immunity | 15.17 | 20 | 10 |
| All restrictions lifted in Northern provinces | 22.21 | 30 | 15 |
| Direct family members from other households can have social contact | 10.30 | 15 | 6 |
| Re-open hospitality and entertainment industry | 19.02 | 25 | 15 |
