# Supplementary material for: Public participation in crisis policymaking. How 30,000 Dutch citizens advised their government on relaxing COVID-19 lockdown measures
Source: PLoS One. 2021 May 6;16(5):e0250614. doi: 10.1371/journal.pone.0250614 (PMC8101923; doi:10.1371/journal.pone.0250614)
Supplement: S6 Appendix — (DOCX) [file pone.0250614.s006.docx]

**S6 Appendix: Corrected optimal portfolio for representative education level groups**

Dekker et al. (2019) provides a general expression of the expected utility of a portfolio for different sociodemographic groups. Let $g=1,\ldots,G$ be a sociodemographic group of the population with its own expected utility equal to $EU_{g}$. Then the expected utility of society is the weighted sum of the expected utility of each sociodemographic group:

$$EU_{corrected}=\sum_{g=1}^{G} Q_{g}EU_{g}=\sum_{g=1}^{G} Q_{g}\left( y_{0}E\left[ \Psi_{ng0} \right]+\sum_{j=1}^{J} y_{nj}E\left[ \Psi_{ngj} \right] \right)$$

Where $Q_{g}$ represents the proportion of individuals that belong to sociodemographic group $g$. Notice that $\Psi_{ng0}$ depends of each sociodemographic group, as a difference with the expression of section 4.3, which is independent of $g$. This implies that a corrected optimal portfolio requires different parameter estimates of the MDCEV model for the different sociodemographic groups. Then, the computation of a corrected optimal portfolio involves the evaluation of $EU_{corrected}$ for all feasible combinations of policy options (i.e. the combinations that satisfy the resource constraint), and values for $Q_{g}$ taken from external sources, such as census data.

S6 Table 1 summarizes the results of the MDCEV model with a low education effect incorporated to allow the computation of corrected optimal portfolios. These effects are included as additional policy-specific constants present in respondents with low education levels (VMBO, MAVO, Mulo or lower).

S6 Table 1: MDCEV estimation results with low education effects.

|  | **Estimates** | **Low education effects** |
| --- | --- | --- |
| **Policy-specific constants:** |  |  |
| 1: Nursing and care homes allow visitors | 2.7623^***^ | -0.0356 |
|  | (0.0320) | (0.0490) |
| 2: Re-open businesses (other than contact professions and hospitality industry) | 2.6799^***^ | -0.5540^***^ |
|  | (0.0272) | (0.0488) |
| 3: Re-open contact professions | 3.2557^***^ | -0.3297^***^ |
|  | (0.0313) | (0.0527) |
| 4: Young people may come together in small groups | 1.9070^***^ | -0.4114^***^ |
|  | (0.0148) | (0.0493) |
| 5: All restrictions lifted for people with immunity | 1.6116^***^ | 0.1422^**^ |
|  | (0.0274) | (0.0526) |
| 6: All restrictions lifted in Northern provinces | 1.6634^***^ | 0.0845 |
|  | (0.0399) | (0.0580) |
| 7: Direct family members from other households can have social contact | 2.5272^***^ | -0.0311 |
|  | (0.0351) | (0.0502) |
| 8: Re-open hospitality and entertainment industry | 2.7437^***^ | -0.4511^***^ |
|  | (0.0399) | (0.0489) |
| **Taste parameters:** |  |  |
| Additional 10.000 deaths of people of +70 years | -0.5904^***^ |  |
|  | (0.0993) |  |
| Additional 10.000 deaths of people of less than 70 years | -0.9304^**^ |  |
|  | (0.2942) |  |
| Additional 10.000 people with permanent physical injury | -0.1137^***^ |  |
|  | (0.0174) |  |
| Minus 10.000 people with permanent mental injury | 0.0012 |  |
|  | (0.0037) |  |
| Minus 10.000 households that have lost 15% of income | 0.0085^***^ |  |
|  | (0.0025) |  |
| Observations | 24004 |  |
| Log-likelihood | -117305.5958 |  |
| AIC | 234569.1916 |  |
| BIC | 234399.3861 |  |
| **Note:** Standard errors in parenthesis. **Statistical significance:** ^***^p < 0.001, ^**^p < 0.01, ^*^p < 0.05 | | |

S6 Table 2 summarizes the corrected optimal portfolios using the estimates of the MDCEV model and using a correction rate of 28,5% for low educated individuals, according to the Dutch census data. We observe no differences with the uncorrected optimal portfolio of Table 5.

S6 Table 2: Corrected optimal portfolios of relaxation options.

|  | Average | Pessimistic | Optimistic |
| --- | --- | --- | --- |
| 1: Nursing and care homes allow visitors |  |  | X |
| 2: Re-open businesses (other than contact professions and hospitality industry) | X |  | X |
| 3: Re-open contact professions | X | X | X |
| 4: Young people may come together in groups |  |  | X |
| 5: All restrictions lifted for people with immunity |  |  |  |
| 6: All restrictions lifted in Northern provinces |  |  |  |
| 7: Direct family members from other households can have social contact | X |  | X |
| 8: Re-open hospitality and entertainment industry |  |  |  |
| **Added pressure onto the healthcare system** | **32%** | **15%** | **34%** |
