# Supplementary material for: Public participation in crisis policymaking. How 30,000 Dutch citizens advised their government on relaxing COVID-19 lockdown measures
Source: PLoS One. 2021 May 6;16(5):e0250614. doi: 10.1371/journal.pone.0250614 (PMC8101923; doi:10.1371/journal.pone.0250614)
Supplement: S7 Appendix — (DOCX) [file pone.0250614.s007.docx]

**S7 Appendix: Survey Instrument**

**Participatory Value Evaluation about relaxing COVID-19 lockdown measures**

**Instruction part**

Welcome to this online consultation about the relaxation of corona lockdown measures from 20^th^ May – 20^th^ July, 2020.

Following the outbreak of the new coronavirus, COVID-19, in the Netherlands, the government has taken various measures to control the spread of the virus, to protect high-risk groups, such as the elderly and people in a precarious state of health, and to prevent various parts of the healthcare system from becoming overloaded.

Now that the measures appear to be effective, a number of these measures can be relaxed. **Would you like certain measures to be relaxed between 20^th^ May and 20^th^ July? And if so, which measures should be relaxed first?** The government would like to receive advice from a large group of Dutch citizens about which of these ‘relaxation options’ are preferred.

The research is being done by researchers at Delft University of Technology in collaboration with researchers from other universities and researchers from the Dutch National Institute for Public Health and Environment (RIVM). Policy staff from the Ministry of Health, Welfare and Sport and the Ministry of Finance also participated. The results of the research will be shared with the RIVM and other researchers who think along with and advise the government about corona.

We would like to thank you very much, in advance, for participating in this consultation!

This consultation is in two parts:

**Part 1: Advice on the relaxation options (takes: 15 – 20 minutes)**

We present a number of ways in which corona measures can be relaxed over the next two months (‘relaxation options’). Thereafter, we ask you to advise the government. Do you think the government should introduce relaxation options from 20^th^ May – 20^th^ July, 2020, and if so, which relaxation options should be chosen?

**Part 2: Other advice and rationale (takes: 5 – 10 minutes)**

We ask you if there are any relaxation options that you feel should **not** be considered. We then ask you to explain your choices. We are interested to know why you feel that certain measures should or should not be relaxed. Lastly, we ask a number of general questions about you (gender, age, where you live, profession).

**Rules**

- The research has been approved by TU Delft’s Ethics Committee.
- Your answers will be saved at TU Delft on a secure server until no later than 27^th^ April, 2030.
- Only citizens over 18 years old may participate in this research.

**Participating interface: The policy measures**


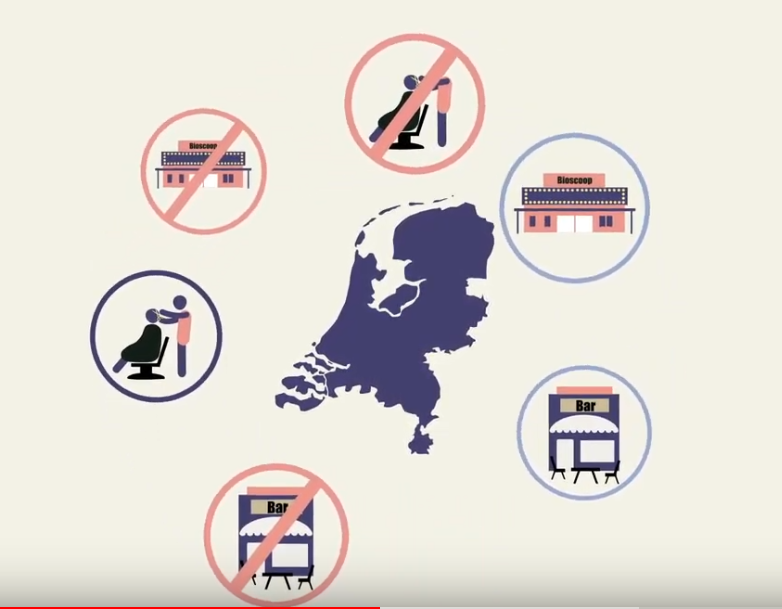

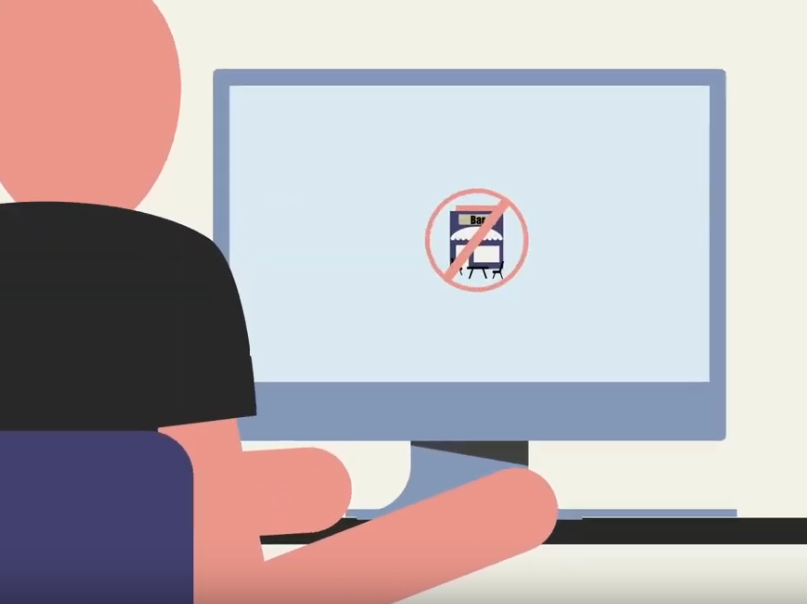


**Nursing and care homes allow visitors**

Care homes allow one visitor 2 times a week. The elderly and people with a mental or physical disability who live in a care home can receive visitors again. Visitors must be healthy.

**The most important positive effects:**

- Because the elderly and people with a mental or physical handicap can receive visitors, there is a reduction in psychological complaints (especially from the people living in care homes, but also from the people who can visit them again).
- For care home staff the situation is much more pleasant as people are not as lonely.

**The most important negative effects:**

- Increase in the mortality rate among care home residents (especially people in the 75+ category). Residents who become ill due to corona are often not sent to Intensive Care, but they die in the care home.
- Higher work pressure for care home workers.

**The effects of this relaxation option:**

Below you will find more information about the effects of this relaxation option. Click here for more information about the uncertainties surrounding the estimations of these effects.

When we talk about impacts, we mean the **differences** between relaxing the measures within two months (20^th^ May – 20^th^ July, 2020) and relaxing the measures after two months (from 20^th^ July, 2020).

**Employees in contact professions (e.g. hairdressers) go back to work**

When you choose this relaxation option, employees in contact professions will be able to work again in the next two months. Hairdressers, beauticians, make-up artists, pedicurists, manicurists, driving instructors and tattoo artists can all re-open their shops. This will be done in stages. For example, hairdressers will be able to re-open their salons before tattoo artists. Employees will have to try to keep contact with their customers to a minimum and should wear as much protective clothing as possible.

Employers can choose to allow staff who fall under a risk category (e.g. people with pulmonary disease, diabetes or a chronic heart condition) to work from home. People are advised to work from home if they have symptoms that could indicate corona (e.g. a runny nose, cough or fever). Public transport may be used but the 1.5 meter distance rule applies here too, so there is a chance of increased travel time.

**The most important positive effects:**

- The economy will start to get moving again. Therefore, the number of bankruptcies and job losses will decrease compared to the situation if these professions are only allowed to re-start after 20^th^ July. This means that there will be fewer people (15%) whose income is badly affected for a period of more than three years.
- Decrease in sustained psychological complaints from people whose psychological complaints were caused by their job or business being at risk.

**The most important negative effects:**

- More people will become ill. This is because the risk of getting infected is relatively high within the contact profession group and because employees in contact professions, in general, are constantly meeting other people. If they are ill, they can infect large groups of people, who in turn spread the virus across the region.

**The effects of this relaxation option:**

Below you will find more information about the effects of this relaxation option. Click here for more information about the uncertainties surrounding the estimations of these effects.

When we talk about impacts, we mean the **differences** between relaxing the measures within two months (20^th^ May – 20^th^ July, 2020) and relaxing the measures after two months (from 20^th^ July, 2020).

**Social contact is allowed again for direct family members from other households**

Currently, only people from within the same household don’t have to keep a 1.5 meter distance from one another. When you choose this relaxation option, physical contact between first and second tier family members will be allowed. For example, grandparents may reestablish contact with their grandchildren, and brothers and sisters can visit one another again. Direct contact with third tier family members (uncles, aunts, cousins, great-grandparents) are still not allowed. Only 50 people are allowed to attend weddings if the 1.5 meter distance rule is respected by everyone who isn’t a direct family member.

**Positive effects:**

- Relaxing this measure will lead to a decrease in loneliness.
- Happy moments (birthdays and weddings) and sad moments (deaths) can be commemorated in a more enjoyable way.

**Negative effects:**

- The number of people that have direct contact (within 1.5 meters) with one another will vastly increase, which means that the number of sick people and the mortality rate will increase. There are two reasons why the number of deaths will still be relatively restricted: 1) Expectations are that, especially at first, many people will put off visiting family members who are in one of the higher risk groups; 2) It will still be the same circle of people who have contact with one another. This ensures that the virus can’t spread as quickly as when people constantly come into contact with a different group of people.

**The effects of this relaxation option:**

Below you will find more information about the effects of this relaxation option. Click here for more information about the uncertainties surrounding the estimations of these effects.

When we talk about impacts, we mean the **differences** between relaxing the measures within two months (20^th^ May – 20^th^ July, 2020) and relaxing the measures after two months (from 20^th^ July, 2020).

**Businesses open again (except the hospitality industry and contact professions)**

At the moment, Dutch citizens have to work from home if at all possible. Therefore, millions of Dutch citizens are currently working from home (consider the employees of large corporates, universities and the civil service). When you choose this relaxation option, employees can return to their offices when it can be guaranteed that they will be able to keep a 1.5 meter distance. Hospitality industry workers and contact professionals (such as hairdressers and physiotherapists) are not included in this relaxation option.

Employers can choose to allow staff who fall under a risk category (e.g. people with pulmonary disease, diabetes or a chronic heart condition) to work from home. People are advised to work from home if they have symptoms that could indicate corona (e.g. a runny nose, cough or fever). Public transport may be used but the 1.5 meter distance rule applies here too, so there is a chance of increased travel time.

**The most important positive effects:**

- The economy will start to get moving again. Therefore, the number of bankruptcies and job losses will decrease compared to the situation if these professions are only allowed to re-start after 20^th^ July. This means that there will be fewer people (15%) whose income is badly affected for a period of more than three years.
- Decrease in the number of sustained psychological complaints caused by working from home. Having to work from home can lead to loneliness or signs of a burn-out, for example.

**The most important negative effects:**

- When the commuter traffic increases, the virus will spread relatively quickly. The number of people who become sick will increase. The number of deaths and the number of sustained physical health problems will also increase because of this.

**The effects of this relaxation option:**

Below you will find more information about the effects of this relaxation option. Click here for more information about the uncertainties surrounding the estimations of these effects.

When we talk about impacts, we mean the **differences** between relaxing the measures within two months (20^th^ May – 20^th^ July, 2020) and relaxing the measures after two months (from 20^th^ July, 2020).

**All restrictions are lifted for people who are immune**

When you choose this relaxation option, people who have had the coronavirus can continue living their normal life without any restrictions. Tests can show if someone has enough antibodies in their blood. When this is the case, citizens will receive a corona letter and they should always carry this around with them, as a kind of passport. In the next two months, these tests will not yet be sufficiently reliable, so it could be that someone who has tested positive, could, in fact, not be immune (due to a flawed positive test) and could become ill through this. At the moment, it appears that approximately 4% of all Dutch citizens have had corona (approximately 650,000 people), but the percentage of Dutch citizens who have built up sufficient antibodies is not yet known.

**Positive effects:**

- People who are immune can again come and go wherever they please. They can go back to work and this means that there will be fewer people with a serious (15%) drop in income for a period of more than three years.
- People who are immune can visit family and friends again. Through this, the number of people who feel lonely will decrease.

**Negative effect:**

- In the next two months, the immunity tests could still be unreliable, so people who appear to be immune could still become infected. Under normal circumstances, it can take years to develop a good immunity test. Also, people who only have a few antibodies in their blood, can probably become infected again, even if they have already had the virus. As a consequence, these people can infect a lot of other people if they have contact with people from across the country. This short film provides more information about the problems when testing for immunity.
- The instructions explained that you should assume that the testing capacity has vastly improved, but the number of people that can be tested per day remains limited. Some people who are immune will have to wait for a long time before they are tested.
- People who aren’t immune could have more problems sticking to the measures now that they see that people who are immune can come and go as they please. The virus will spread quickly when the number of people that no longer adhere to the rules increases, and there will even be people who catch the virus on purpose so that they will be given a corona letter. It is unclear if this measure can be easily regulated.

**The effects of this relaxation option:**

Below you will find more information about the effects of this relaxation option. Click here for more information about the uncertainties surrounding the estimations of these effects.

When we talk about impacts, we mean the **differences** between relaxing the measures within two months (20^th^ May – 20^th^ July, 2020) and relaxing the measures after two months (from 20^th^ July, 2020).

**Hospitality and entertainment sectors open again**

When you choose this relaxation option, the hospitality and entertainment sector (consider restaurants, cafés, gyms, theme parks, museums, theaters and movie theaters) can re-open if it can be guaranteed that people will be able to keep to the 1.5 meter distance rule. The 1.5 meter distance rule applies to staff in relation to one another, as well as to staff in relation to the customers.

Employers can choose to allow staff who fall under a risk category (e.g. people with pulmonary disease, diabetes or a chronic heart condition) to work from home. People are advised to work from home if they have symptoms that could indicate corona (e.g. a runny nose, cough or fever). Public transport may be used but the 1.5 meter distance rule applies here too, so there is a chance of increased travel time.

**The most important positive effects:**

- The economy will start to get moving again. Therefore, the number of bankruptcies and job losses will decrease compared to the situation if these professions are only allowed to re-start after 20^th^ July. This means that there will be fewer people (15%) whose income is badly affected for a period of more than three years.
- Decrease in sustained psychological complaints from people whose psychological complaints were caused by their job or business being at risk. The number of psychological complaints will also decrease because people will be able to enjoy going to a café, restaurant, bar or some other entertainment.

**The most important negative effects:**

- Despite the distancing rule, there will be an increase in the number of people who become ill. It will also be the cause of an increase in mortality rates and the number of sustained physical health problems.

**The effects of this relaxation option:**

Below you will find more information about the effects of this relaxation option. Click here for more information about the uncertainties surrounding the estimations of these effects.

When we talk about impacts, we mean the **differences** between relaxing the measures within two months (20^th^ May – 20^th^ July, 2020) and relaxing the measures after two months (from 20^th^ July, 2020).

**Restrictions lifted in Friesland, Groningen and Drenthe**

When you choose this relaxation option, all the restrictive measures will be lifted for the provinces of Friesland, Groningen and Drenthe because the coronavirus is very much under control in these regions. Citizens in these provinces account for just 3% of the number of hospital admissions through corona in the Netherlands. There are two exceptions. The restrictive measures will not be lifted for people in high risk groups (the elderly over 75, and people with pulmonary disease, diabetes or a chronic heart condition). For the next two months, events and meetings with more than 50 attendees will still be forbidden. Hospitals across the country will accommodate any patients from these regions.

In the first few weeks, people from these Northern provinces may only enter or leave the area if they have a valid reason. Checks will be held on the access roads. This can restrict the spread of the virus to other regions and it will restrain any ‘hospitality tourism’. Depending on how the pressure on the healthcare system is affected, this measure could be relaxed or tightened. The percentage shown at the top of this page is the target rate for pressure on the healthcare system.

**The most important positive effects:**

- The economy in Friesland, Groningen and Drenthe will start to get moving again. There will be limited economic damage caused by the corona crisis in this region.
- Fewer complaints of loneliness and other physical complaints, such as a burn-out and depression among the citizens of Friesland, Groningen and Drenthe.

**The most important negative effects:**

- The number of infected people, deaths, and cases of sustained physical health problems will increase, but because the testing capacity will have greatly increased, people who are infected can be quickly isolated.
- People who live in the other provinces, outside Friesland, Groningen and Drenthe, will have more problems sticking to the measures now that they see that people in another part of the Netherlands can come and go as they please. The virus will spread quickly when the number of people that no longer adhere to the rules increases. It is unclear if this can be easily regulated.
- The checks on the access roads will create increased travel time.

**The effects of this relaxation option:**

Below you will find more information about the effects of this relaxation option. Click here for more information about the uncertainties surrounding the estimations of these effects.

When we talk about impacts, we mean the **differences** between relaxing the measures within two months (20^th^ May – 20^th^ July, 2020) and relaxing the measures after two months (from 20^th^ July, 2020).

**Young people may meet up in groups**

Currently, young people up to 18 years of age are allowed to take part in organized sport. When you choose this relaxation option, the corona measure will be lifted in phases between 20^th^ May and 20^th^ July for young people up to and including 25 years old, in that they will be allowed to meet in groups and they will no longer have to respect the 1.5 meter distance rule. It is very important that they **do** have to respect the 1.5 meter distance rule for older people and this will be upheld.

Young people carry a much smaller risk of becoming seriously ill from the coronavirus than the elderly (although there are exceptions). Just 1% - 1.5% of corona patients that have been admitted to hospital were under 25 years old.

In the first phase, children under 12 years old can meet up again in groups of 10. They can do contact sports in a group of 10, for example. If this appears to have little effect on the number of corona infections, and it appears that young people still respect the 1.5 meter distance rule when they are with older people, then the relaxation of the measure will be gradually phased in and extended to bigger groups (25, 50) and to older age groups (12 – 18 years old; 18 – 25 years old). So, in an ideal situation, by the end of June, Dutch citizens who are 25 years old or younger will be able to meet up again in groups of 50, but it could also be that it is kept to the under 12s in groups of no more than 10 for quite a while.

Contact between young people and the elderly must still always be avoided, of course.

**The most important positive effects:**

- There will be a decrease in sustained psychological complaints among young people. Also, there will be more breathing space within families now that the children can meet up with their friends again.

**The most important negative effects:**

- There will be a slight increase in the number of young people admitted to hospitals and the mortality rate, but this will be relatively limited because young people carry a much lower risk of becoming seriously ill from the coronavirus. People who have contact with young people will assume a relatively big risk.
- It will become more difficult for young people to respect the 1.5 meter distance rule between themselves and older people when they no longer have to respect this rule among themselves.

**The effects of this relaxation option:**

Below you will find more information about the effects of this relaxation option. Click here for more information about the uncertainties surrounding the estimations of these effects.

When we talk about impacts, we mean the **differences** between relaxing the measures within two months (20^th^ May – 20^th^ July, 2020) and relaxing the measures after two months (from 20^th^ July, 2020).

**You can compare your selected relaxation options.**


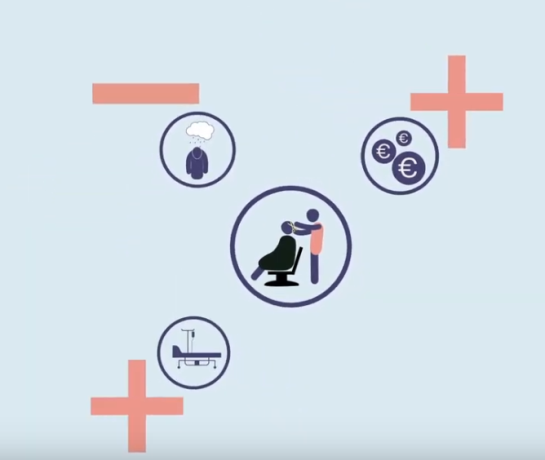

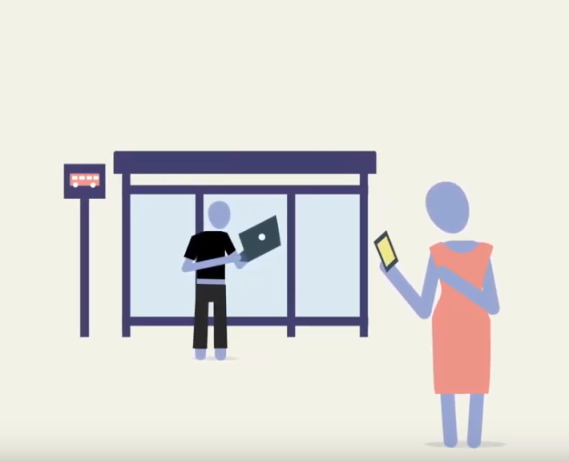


Comparison options:

- Increased pressure on the healthcare system
- Increase in the number of deaths 70+
- Increase in the number of deaths under 70 years old
- Increase in the number of people with permanent physical health problems
- Decrease in the number of people with permanent psychological health problems
- Decrease in the number of households with long-term loss of income

**Screenshot of the webtool (In Dutch)**


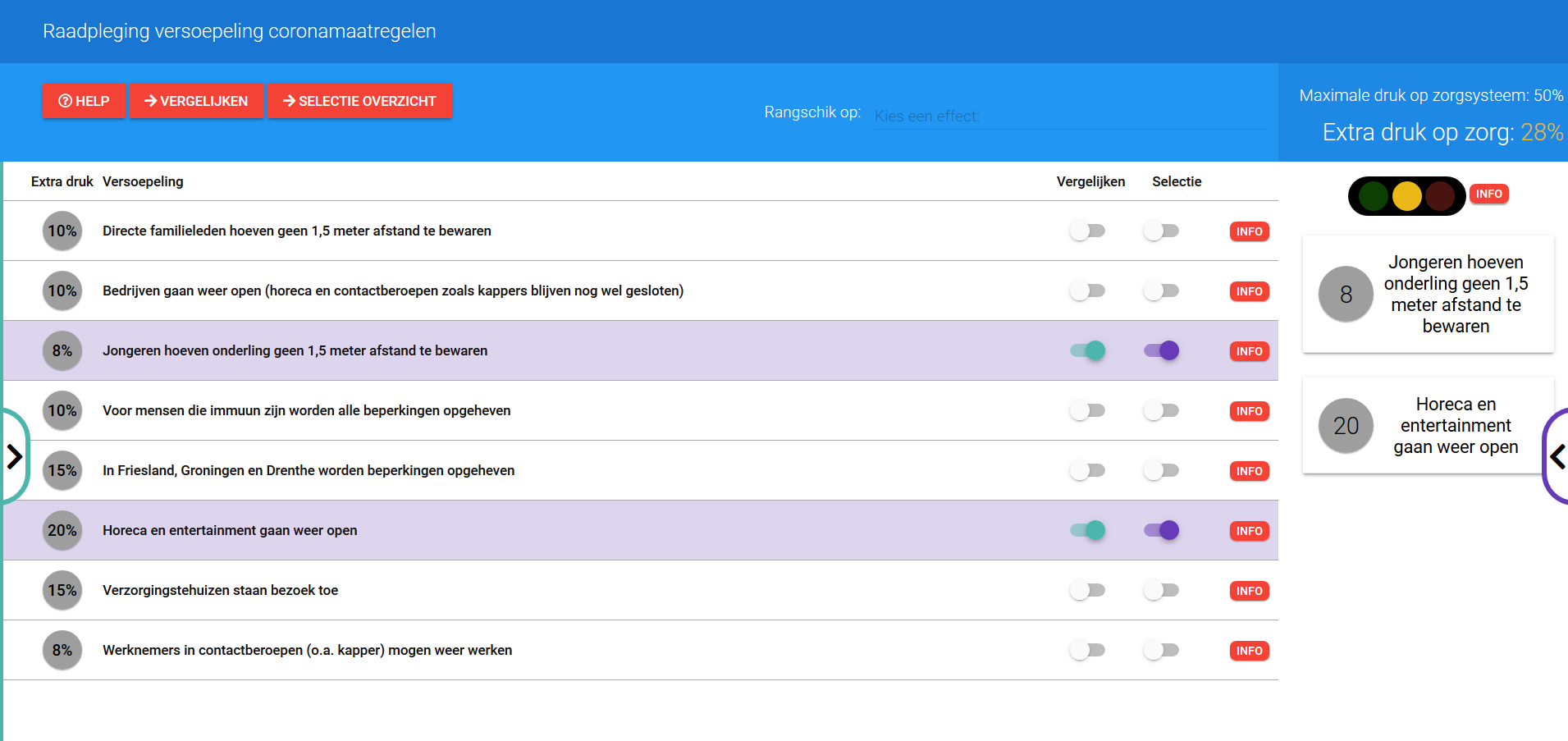


**You have chosen these relaxation options.**

Below you can see the relaxation options that you have recommended.

In this survey, you can only recommend a limited number of relaxation options. The pressure on the healthcare system may not increase by more than 50%. You may recommend that the government should not relax any measures between 20^th^ May and 20^th^ July.

If you would like to amend your advice, click on the red ‘back’ button. If you are happy with your advice, click on the ‘send’ button below. We will then ask you a few more brief questions.

**Pressure on the healthcare system**

**Green (0% - 25% increased pressure)**

The healthcare system is not overstretched. People in the healthcare sector do not have to work overtime. The healthcare sector can catch its breath, and the chance of employees leaving the healthcare sector in the short and long term is more or less the same as in the period before the corona crisis (at the start of 2020). There is sufficient room to be able to handle treatments other than corona.

**Yellow (26% - 40% increased pressure)**

The healthcare system is overstretched. People in the healthcare sector work an extra 6 hours per week, on average. The healthcare sector can catch its breath somewhat but there is still a chance that employees in the healthcare sector will leave in the short and long term. Some of the treatments other than corona have to be postponed.

**Red (41% - 50% increased pressure)**

The health care system is heavily overstretched. People in the healthcare sector work an extra 12 hours per week, on average. The healthcare sector is unable to catch its breath and there is a big chance that employees in the health care sector will leave in the short and long term. All treatments other than corona that are not absolutely necessary have to be postponed. There is a possible shortage of protective material.

Nurses and doctors who would normally work on another ward (e.g. Oncology, Cardiology and Neurology) must now work on the corona intensive care ward. So, in fact, for a long time, these healthcare workers will have to do a different job. This can be tough as healthcare workers will have to work on a different team, so there could be some doubt about if the right choice has been made.

**An increase in pressure of more than 50% is not possible**

In this survey it is not possible to choose an increase in pressure on the healthcare system of more than 50%, because it would then no longer be possible to treat all patients that would have a chance of recovery. The government wants to avoid this scenario.

**Part 2: Additional questions**

We would like to ask you a few further, general questions.

1. Please motivate your choice (participants are asked to provide verbal explanations for the options they selected):

1. Are there any relaxation options that you think the government should not consider?
2. Can you explain why you feel that these relaxation options should not be considered?
3. Can you indicate which of the relaxation options would have a big effect on your life?

For each policy option:

- No effect
- Small effect
- Reasonable effect
- Big effect
- Very big effect

1. As well as consulting a large group of citizens, the government will also be consulting a number of researchers. In your opinion, how much value should the government put on this advice from the citizens and the researchers?

- Only follow the advice of citizens
- More value to advice of citizens than academics
- Equal value to advice of citizens and academics
- More value to advice of academics than citizens
- Only follow the advice of academics

1. Could you explain your answer to the previous question?
2. You will now be shown a number of statements. For each statement, please indicate how strongly you agree or disagree with this statement?

I am certain that my advice is right

- Totally disagree
- Disagree
- Neutral
- Agree
- Totally agree

By taking part in this research I have learned more about the choices that the government has to make

- Totally disagree
- Disagree
- Neutral
- Agree
- Totally agree

Participating in this research has influenced by opinion about the appropriateness of certain relaxation options

- Totally disagree
- Disagree
- Neutral
- Agree
- Totally agree

This is a good way of involving Dutch citizens in decisions that the government has to make about relaxing corona measures between 20^th^ May and 20^th^ July

- Totally disagree
- Disagree
- Neutral
- Agree
- Totally agree

The government should use this method more often to include citizens in government policy making.

- Totally disagree
- Disagree
- Neutral
- Agree
- Totally agree

Now that the government has asked for my advice, I am more inclined to comply with the corona measures

- Totally disagree
- Disagree
- Neutral
- Agree
- Totally agree

I am confident that most Dutch citizens will adhere to the corona measures in the next three months

- Totally disagree
- Disagree
- Neutral
- Agree
- Totally agree

Because the government is involving citizens in this way, it will be easier for me to accept the government’s final decision concerning the relaxation of corona regulations between 20^th^ May and 20^th^ July

- Totally disagree
- Disagree
- Neutral
- Agree
- Totally agree

1. Would you like to pass on any ideas to the government for when they are considering the relaxation of the corona measures? Include your advice below:
2. Have you been infected by the corona virus?

| No, tested and negative |  |
| --- | --- |
| Probably not, but haven’t been tested |  |
| Probably, but haven’t been tested |  |
| Yes, tested and positive |  |
| I don’t want to answer this questions |  |

1. Are there people in your direct environment (family in your household, other family, friends) who are (or have been) infected by the coronavirus?

| No, tested and negative |  |  |
| --- | --- | --- |
| Probably not, but haven’t been tested |  |  |
| Probably, but haven’t been tested |  |  |
| Yes, tested and positive |  |  |
| I don’t want to answer this questions |  |  |

1. How would you estimate the following risks for yourself?

Getting infected with the coronavirus

- Low risk
- Reasonable risk
- High risk
- Extremely high risk

Becoming very ill after being infected by the coronavirus

- Low risk
- Reasonable risk
- High risk
- Extremely high risk

Having to be admitted to hospital after being infected by the coronavirus

- Low risk
- Reasonable risk
- High risk
- Extremely high risk

Dying through being infected by the corona virus

- Low risk
- Reasonable risk
- High risk
- Extremely high risk

1. How do you estimate the risk for at least one person in your direct environment (family in your household, other family, friends):

- Low risk
- Reasonable risk
- High risk
- Extremely high risk

Becoming very ill after being infected by the coronavirus

- Low risk
- Reasonable risk
- High risk
- Extremely high risk

Having to be admitted to hospital after being infected by the coronavirus

- Low risk
- Reasonable risk
- High risk
- Extremely high risk

Dying through being infected by the corona virus

- Low risk
- Reasonable risk
- High risk
- Extremely high risk

1. How old are you?

| 18 - 25 years old |  |
| --- | --- |
| 26 - 35 years old |  |
| 36 - 45 years old |  |
| 46 - 55 years old |  |
| 56 - 65 years old |  |
| 66 - 74 years old |  |
| Above 75 years old |  |

1. What is you highest level of education?

| No formal education |  |
| --- | --- |
| Junior school/primary education |  |
| Lower Vocational Education (trade school, domestic science school, lower technical school, lower economics and admin education, etc.) |  |
| Pre-vocational Secondary Education / Advanced Primary Education (MAVO, VMBO, MULO) |  |
| Higher General Secondary Education / Preparatory Academic Education (HAVO/VWO) |  |
| Secondary Vocational Education (MBO) |  |
| Higher Vocational Education (HBO) |  |
| University |  |

1. What is your current living arrangement?

| I live alone |  |
| --- | --- |
| I live with my partner |  |
| I live with my partner and child(ren) |  |
| I live with a child / children |  |
| I live with roommates |  |
| Others |  |

1. Which province do you live in?

| Groningen |  |
| --- | --- |
| Friesland |  |
| Drenthe |  |
| Overijssel |  |
| Flevoland |  |
| Gelderland |  |
| Utrecht |  |
| North-Holland |  |
| South-Holland |  |
| Zeeland |  |
| North-Brabant |  |
| Limburg |  |

1. What is the net monthly income of your household? This is the total amount from salary, benefits, grants and pensions that your household receives every month.

| Less than 1000 Euros |  |
| --- | --- |
| Between 1000 and 2000 Euros |  |
| Between 2000 and 3000 Euros |  |
| Between 3000 and 4000 Euros |  |
| Between 4000 and 5000 Euros |  |
| Between 5000 and 6000 Euros |  |
| Between 6000 and 7000 Euros |  |
| More than 7000 Euros |  |
| I would rather not answer this question |  |
| I don’t know |  |

1. How do you expect your household’s net income to change in 2020?

| I expect our net income to strongly decrease |  |
| --- | --- |
| I expect our net income to decrease |  |
| I expect our net income to remain the same |  |
| I expect our net income to increase |  |
| I expect our net income to strongly increase |  |

1. What is your current work situation (more than one answer is possible)?

- Paid work on a fixed contract
- Paid work on a temporary contract
- Freelancer / business owner
- Voluntary worker
- Pensioner
- Out of work / looking for work
- Unfit for work
- Receiving welfare benefits
- Housewife / House husband
- I am following a course / studying
- None of the above

1. What is your profession? (only answer this question if you chose one of the top three options listed above as an answer to the previous question)
2. What is your current work situation like? (only answer this question if you filled in a profession for the previous question)

| My work is currently at a standstill |  |
| --- | --- |
| Currently, I have less work |  |
| My work continues as normal |  |
| I currently have more work |  |

1. What did you feel were the strong points about this method?
2. What do you think could be done to improve this method?

**Thank you**

Thank you very much for taking part in this consultation!

If you would like more information about this consultation and the method that we use for these consultations, visit www.tudelft.nl/covidexit/. On this website we also publish the results of this research.

You can send any feedback about this research by email to n.mouter@tudelft.nl
